# The somatotroph pituitary gland function in high-aged multimorbid hospitalized patients with IGF-I deficiency

**DOI:** 10.1007/s11102-024-01406-y

**Published:** 2024-05-31

**Authors:** Olivia Tausendfreund, Martin Bidlingmaier, Sebastian Martini, Hannah Reif, Michaela Rippl, Katharina Schilbach, Ralf Schmidmaier, Michael Drey

**Affiliations:** 1 Department of Medicine IV, University Hospital, LMU Munich, München, Germany; 2 Deggendorf Institute of Technology, Deggendorf, Germany

**Keywords:** GH, IGF-I, Pituitary gland, Somatotropic axis, Somatopause, Aging

## Abstract

**Purpose:**

It is unclear whether the age-related decline in the somatotropic axis stems from a reduced growth hormone (GH) production in the pituitary gland, or from a peripheral origin akin to an acquired GH resistance. With the help of a GHRH/arginine test, high-aged multimorbid hospitalized patients with IGF-I deficiency are to be tested to determine whether there is primarily a pituitary GH deficiency in the sense of a somatopause.

**Methods:**

Seventeen multimorbid patients (eleven men and six women) with a mean age of 82 years, with IGF-I concentrations below two standard deviations of 30-year-old men and women were identified. Patients suffered from a variety of common age-related stable diseases including coronary artery disease, chronic liver or kidney disease, chronic heart failure as well as acute conditions e.g., urosepsis or endocarditis. To assess the somatotropic axis they underwent a GHRH/arginine test. Results were evaluated using descriptive statistics.

**Results:**

In average, the peak concentration of GH after stimulation was 14.8 µg/L with a range from 2.76 to 47.4 µg/L. Taking into account both, gender and BMI (with a mean of 26.5 kg/m²) for each participant, the pituitary gland was adequately stimulated in 16 out of the 17 patients. No patient reported common side effects related to the GHRH/arginine test.

**Conclusion:**

The somatotroph pituitary gland retains its secretory capacity in the advanced aged. Therefore, age does not seem to be the driving pacemaker for the functional decline of the somatotropic axis within the aged population.

## Introduction

Despite its name suggesting a focus solely on growth, growth hormone (GH) plays a crucial role that extends beyond adolescence and attainment of adult height. GH is essential for regeneration and maintenance of tissues throughout the whole body and entire life span. It has broad influence on various metabolic processes, balancing lipolysis and lipogenesis, glucose haemostasis, erythropoiesis, protein synthesis, as well as bone and muscle metabolism [1]. Through activation of its mediator insulin-like growth factor I (IGF-I), GH also indirectly induces gonadal proliferation, the formation of neuronal synapses, promotes cardiac cell proliferation and has anti-inflammatory effects on the vasculature [2–5]. A prevailing consensus suggests that GH production in the pituitary gland diminishes with advanced age, a phenomenon commonly referred to as somatopause [1, 6, 7]. This age-related state of deficiency in GH and IGF-I might contribute to various common moderate changes with advanced age in body composition, activity levels, cognition, and metabolism [7–10]. However, when IGF-I concentrations fall below the age-appropriate range, these changes might be exacerbated and similar to a GH deficiency in the traditional sense and have unfavorable influence on the development of diseases like osteoporosis, sarcopenia or dementia. Interestingly, our own data indicate a different finding. Previous data from our group showed that in hospitalized patients suffering from sarcopenia, a common age-related loss of muscle mass and muscle strength, IGF-I concentrations were decreased, while GH concentrations were significantly elevated [11]. Both, high GH and low IGF-I serum concentrations in this cohort were interpreted as an acquired growth hormone resistance, which might be the origin of sarcopenia in this group of patients [11]. Consequently, this represents a different entity of imbalance in the somatotropic axis and may also entail different potential therapy approaches compared to a GH deficit in the classical sense.

To confirm or dismiss the classical somatopause hypothesis, a precise analysis using the GHRH/arginine test in aged patients with IGF-I deficiency is therefore urgently need.

## Methods

### Participants

All patients from the geriatric day clinic and acute geriatric ward of the LMU University Hospital Munich, Germany, have the opportunity to voluntarily provide their data for a comprehensive patient registry on sarcopenia and its association with other geriatric diseases. Between November 2022 to September 2023, 17 individuals from the pool of registered patients were identified as IGF-I deficient, falling below two standard deviations (-2.0SD) of 30-year-old men and women in IGF-I concentrations (cut-off concentrations of 86 µg/L for men and 67 µg/L for women) [12]. Subsequently, these patients underwent dynamic testing of the somatotropic axis, in our cases a standardized GHRH/arginine test [13–15]. None of the included patients had shown other signs of pituitary insufficiency due to a decline in other axes of the pituitary gland in the past, neither had they undergone endocrinological treatment for pituitary insufficiency.

The Ethics Committee of the Medical Faculty of the Ludwig-Maximilians-University has approved the registry inclusion (Ethical vote no.17–874) as well as the study protocol underlying this manuscript (Ethical vote no. 23–0981).

### GHRH /arginine test

Baseline concentrations for both, GH and glucose were determined at -15 min and 0 min. Subsequently, an intravenous injection of 100 µg GHRH (GHRH Ferring) was administered within one minute, concomitant with an Arginine infusion of 500 ml over 30 min (L-Arginine hydrochloride 6%). GH and glucose concentrations were measured at 30, 60, 90, and 120 min to monitor the dynamic changes in response to the stimulation [16].

### Laboratory measurements

GH serum concentrations were measured by an automated chemiluminescence immunoassay (CLIA, IDS- iSYS; Immunodiagnostic Systems, Boldon, UK). The assay used had analytical and functional sensitivities of 0.01 µg/L and 0.04 µg/L, with a dynamic range from 0.04 to 100 µg/L [17].

For the individual IGF-I values, an automated chemiluminescence immunoassay (CLIA, IDS-iSYS; Immunodiagnostic Systems, Boldon, UK), which had a limit of detection of 4.4 ng/mL and limits of quantification of 8.8 ng/mL was used. The dynamic range here was 10–1200 ng/mL [12].

To enhance the accuracy of the analysis, the measured GH serum concentrations from the GHRH/arginine tests were evaluated in a BMI and gender-adjusted manner, following the normative data developed for the used immunoassay [13]. In detail, this meant that for male patients with a BMI below 25 kg/m², a cutoff of 6.5 ng/ml applied, for a BMI between 25 to under 30 kg/m², a cutoff of 3.5 ng/ml applied, and for patients over 30 kg/m², a cutoff of 2.2 ng/ml GH applied. Correspondingly, for female patients, cutoffs of 9.7 ng/ml, 8.5 ng/ml, and 4.4 ng/ml applied.

### Additional measures

#### Mini Nutritional Assessment Short Form (MNA-SF)

The short version of the MNA was used to evaluate the nutritional status [18]. The assessment encompasses questions about food intake (0–2 points), weight loss (0–3 points), mobility (0–2 points), acute illnesses and mental stress (0–2 points) and neuropsychological problems (0–2 points) in addition to the patient`s BMI (0–3 points). The maximum attainable score was 14 points. Scores of 14 − 12 points were considered indicative of a normal nutritional status, 11 − 8 points suggested a risk of malnutrition, and scores from 7 − 0 points indicated malnutrition.

#### Activities of Daily Living (ADL)

As a tool to assess an individual’s ability to perform basic activities necessary for daily life, the ADL Index was used [19]. The index score ranged from 0 to 100 points, with higher scores indicating greater independence in performing these activities, such as grooming, dressing and mobility.

Additionally, to this, the current number of diagnoses written in any medical report and the current number of medications for each patient were determined.

### Statistics

Metric variables are reported as means along with their standard deviations and all statistic calculations were performed using SPSS (IBM SPSS Statistics for Windows).

## Results

### Baseline characteristics

Table 1 shows the baseline characteristics of the participants. The mean age of the patients was 82 years, the average BMI was 26.5 kg/m^2^. Five patients were enrolled from the day clinic, 13 patients from the acute geriatric ward. The participants suffered from various comorbidities, including stable chronic (e.g., coronary artery disease, osteoporosis, sarcopenia or chronic heart, liver and kidney disease) and acute conditions (e.g., urosepsis, spontaneous pneumothorax, endocarditis, SARS-CoV2 infection or acute kidney failure); Table 2 gives a short overview of the individual diagnoses leading to admission to the geriatric day clinic and acute geriatric ward.

With an average of 8 points on the Mini Nutritional Assessment, most of the patients were at least at risk of malnutrition at the time of testing. Moreover, with an ADL index of 60, patients were on average classified as dependent on assistance.

The average IGF-I concentration was 54 µg/L. Therefore, all patients not only exhibited concentrations below two standard deviations of a 30-year-old, but also lower levels than the 50% percentiles for individuals of the 82-year-old age group, regardless of gender [12].

Mean basal plasma GH level was 1,37 µg/L. Our laboratory measurements did not detect any deficits in the hormones of the other axes secreted from the anterior pituitary lobe. 5 out of 17 patients were taking thyroid medication at the time of testing, none of the patients supplemented oestrogen or biotin. As expected, the only significant difference between genders was found in serum testosterone concentration.

**Table 1:** Characteristics of the participants at baseline

	Total (*n* = 17)	Female (*n* = 6)	Male (*n* = 11)	*P*-value	Patient # 7
***Clinical characteristics***					
Age (years)	82 (6) [72–93]	81 (5) [74–85]	83 (7) [72–93]	0.416	75
BMI (kg/m^2^)	26.5 (6.3) [15.4–36.1]	25.3 (7.1) [15.4–35.2]	27.1 (6.1) [18.3–36.1]	0.609	28.2
Number of medications	11 (3) [6–15]	11 (4) [6–15]	12 (3) [7–15]	0.528	14
Number of diseases	12 (5) [5–27]	10 (3) [7–14]	12 (5) [5–27]	0.192	12
MNA-SF Score	8 (3) [5–13]	9 (3) [5–13]	7 (2) [5–12]	0.396	7
ADL Index	60 (29) [10–100]	69 (30) [35–100]	55 (29) [10–95]	0.389	25
***Laboratory measurements***					
GH (µg/L)	1.37 (2.63) [0.14–11.35]	2.45 (4.37) [0.36–11.35]	0.79 (0.68) [0.14–2.20]	0.396	2.20
IGF-I (µg/L)	54 (17) [10–83]	45 (19) [10–62]	58 (16) [29–83]	0.174	46
TSH (µU/ml)	2.26 (0.31) [0.10–5.23]	1.79 (1.50) [0.10–4.20]	2.52 (1.80) [0.20–5.23]	0.391	5.23
fT4 (ng/dl)	1.4 (0.3) [0.8-2.0]	1.5 (0.3) [1.2-2.0]	1.3 (0.3) [0.8–1.7]	0.168	1.6
ACTH (pg/mL)	17 (12) [4–48]	16 (17) [4–48]	17 (9) [7–34]	0.907	35
Cortisol (µg/dl)	16.7 (8.0) [6.5–40.9]	16.6 (4.6) [9.7–23.2]	17.0 (12.5) [6.5–40.9]	0.942	15.9
LH (U/l)	13.9 (18.2) [0.1–64.9]	19.6 (29.4) [0.1–64.9]	10.8 (8.4) [0.1–26.1]	0.501	4.4
FSH (U/l)	24.4 (31.0) [2.2–127.0]	39.2 (47.8) [2.2–127.0]	16.3 (13.9) [2.3–48.6]	0.298	5.4
Estradiol (pg/ml)	47.0 (78.6) [0.1–336.0]	18.0 (21.7) [5.0–61.0]	62.8 (94.2) [0.1 -3 36.0]	0.158	25.1
Testosteron (ng/dl)	266 (259) [28–848]	45 (7) [14, 35, 38–49]	327 (261) [28–848]	0.005*	83
Prolaktin (µg/L)	16.7 (12.5) [6.6–48.9]	18.5 (9.6) [8.8–32.5]	15.7 (14.2) [6.6–48.9]	0.634	8.2
CRP (mg/dl)	3.6 (4.9) [0.1–14.8]	2.9 (5.3) [0.1–13.7]	3.9 (4.9) [0.1–14.8]	0.713	1.5
GFR (ml/min)	64 (23) [23–89]	71 (24) [28–89]	60 (22) [23–88]	0.397	83.0

All measures are presented as mean (SD) [range], p-values for students t-test, *p < 0.05

**Table 2:** Overview of the 17 patients and their individual admission diagnoses

Patient	Age	Gender	Admission Diagnosis
	(years)		
1	81	male	Unsteadiness in gait and tendency to fall
2	83	male	Acute kidney failure
3	93	male	Acute kidney failure
4	77	male	Spontaneous pneumothorax in emphysema, pneumonia
5	89	male	Endocarditis and spondylodiscitis
6	90	male	Chronic kidney insufficiency, spondylarthrosis
7	75	male	Ischemic cardiomyopathy
8	89	male	Acute kidney failure, e. coli bacteraemia
9	81	male	Urosepsis and pyelonephritis, SARS-CoV2 infection
10	72	male	Amputation due to tissue infection
11	83	male	Coronary artery disease
12	74	female	Urinal infection with acute kidney failure
13	85	female	Unsteadiness in gait and tendency to fall, steatosis hepatitis
14	79	female	Atypical pneumonia with acute kidney failure
15	77	female	Chronic dizziness and unsteadiness in gait
16	84	female	Atrial fibrillation
17	85	female	Acute liver failure in chronic liver cirrhosis

### GHRH/arginine test

The mean GH peak after stimulation in the GHRH/arginine test was 14.8 µg/L, ranging from 2.76 µg/L to 47.4 µg/L. 16 out of 17 participants (94%) showed physiologic results, indicating a responsive pituitary gland (Table 3). Baseline data of the male patient number 7, who was none responsive are displayed in Table 1.

None of the patients reported common stimulation test related side effects, such as facial flushing, nausea, headache or transient dysgeusia [20].

**Table 3:** Individual GHRH/arginine test results

Patient	Age	Gender	BMI	IGF-I	Peak GH level	GHRH/arginine test
	(years)		(kg/m^2^)	(µg/L)	(µg/L)	+ / -
1	81	male	18.3	75	29.3	+
2	83	male	20.8	66	14.5	+
3	93	male	21.8	56	12.9	+
4	77	male	22.0	43	12.6	+
5	89	male	25.1	29	11.7	+
6	90	male	26.6	54	25.8	+
7	75	male	28.2	46	2.76	-
8	89	male	31.2	71	4.32	+
9	81	male	33.4	51	5.66	+
10	72	male	34.6	69	8.58	+
11	83	male	36.1	83	14.6	+
12	74	female	15.4	55	11.3	+
13	85	female	21.6	62	14.2	+
14	79	female	21.7	49	10.3	+
15	77	female	35.2	56	5.84	+
16	84	female	28.1	38	20.4	+
17	85	female	29.7	10	47.4	+

(+) = adequate GH response, (-) = inadequate GH response

## Discussion

In our study, 16 out of 17 high-aged multimorbid hospitalized patients with IGF-I deficiency exhibited a physiological GHRH/arginine test result.

Patient number 7, who did not achieve an adequate GH response, did not majorly differ from the other patients based on our observed measurements. One notable difference is that the patient has the highest TSH concentration compared to the other patients. However, there is consensus in the literature that TSH concentration tends to increase with age, hence it broader ranges apply. Therefore, this concentration can be considered as “high-normal” and thus not require treatment [21–23].

Another noticeable parameter characterizing this patient is the lowered testosterone level, common in very sick patients, which aligns with the hospitalization due to ischemic cardiomyopathy with severely impaired cardiac function [24]. Furthermore, it is important to note that the patient had history of stroke, likely contributing to the decline in response during the GHRH/arginine test. Unfortunately, the patient experienced another embolic incident and passed away a few months after the hospital stay, which prevented further investigations.

### The somatopause: an aged and insufficient pituitary gland?

To estimate the secretory capacity of the pituitary gland, measuring spontaneous serum GH concentrations provides only limited information due to its pulsatile release and broad range of influencing stimuli [25]. Nonetheless, it is notably that the GH concentrations of our participants were, on a general notion, not significantly lower but rather higher compared to average GH concentrations in previously published studies on healthy aged patients [26, 27]. To reliably detect a possible GH deficiency, we chose the GHRH/arginine test, which has been shown to be well tolerated with a with a low side-effect profile, especially concerning the cardiovascular risk [16, 28].

While the assessment of the somatotropic axis with the GHRH/arginine test has been conducted in physically trained healthy aged participants of both sex and alone in healthy aged men before, the participants of our study are, to the best of our knowledge, the first high-aged, multimorbid hospitalized participants, aged up to 93 years [26, 27]. Our cohort of participants suffered from a diverse range of common age-related diseases to varying extents, making it a good representative sample of the aged and multimorbid demographic cohort [29]. The applied cut-offs were specifically developed for the exact immunoassay utilized in the analysis of the serum samples, but our data were in line with the results from other publications, as well [13, 20, 30]. Therefore, contrary to the widely held assumption that growth hormone production in the pituitary gland generally decreases with advanced age, our findings challenge this notion as 96% of our patients demonstrated that their somatotroph pituitary gland maintains its activity well into advanced age leading us to assert that the somatopause cannot solely be attributed to a GH-deficient state or might be a myth itself.

### Alternative explanations for an age-related IGF-I deficiency

At this point it is crucial to note that combined GHRH/arginine test achieves the maximum secretory capacity upon external stimulation [13]. The GHRH test itself cannot provide substantial information about a hypothalamic stimulus reduction with age, neither effectively detect it [28]. And although the stimulation originating from the hypothalamus may not necessarily decrease in frequency with age, it does exhibit, at the very least, a reduction in pulse amplitude and duration, as demonstrated in healthy aged subjects previously [31–33]. Consequently, the observed IGF-I deficiency in our patients may be linked to limited hypothalamic activity. This presents an additional dimension to GH regulation, which we cannot assess with our data set. The absence of a comprehensive GH profile, collected throughout the day, leaves the extent of hypothalamic influence on the participants IGF-I deficiency open for further investigation at this point.

On the other hand, the low IGF-I concentrations in our aged patients, whilst having normal to high basal GH concentrations and a normal secretion capacity, can also give hint of a prevailing age-related GH resistance state, we want to explore further in this section.

When discussing GH resistance, it’s essential to differentiate between primary GH resistance and acquired GH resistance. The former, is a rare condition typically manifesting in childhood or adolescence with growth retardation [34]. As all our study participants attained normal height and development, we can exclude this syndrome as a possible origin for the observed low IGF-I within this study cohort.

Alternatively, the age-related decline of IGF-I in our aged patients seems to be more attributable to other peripheral factors, resembling an acquired GH resistance - a concept acknowledged by others before [26, 35, 36]. While deficiencies of the anterior pituitary gland and primary GH resistance are well studied, relatively little is known about the biochemical development of acquired or secondary GH resistance. But besides this lack of knowledge on the exact etiology of the syndrome, a broad range of acute and chronic organ dysfunctions with various pathomechanisms, have already been proven to be associated with an acquired GH resistance [36]. For instance, as circulating IGF-I is mainly synthesized by the liver, diseases such as liver cirrhosis, are reported to be linked to high GH levels with low IGF-I, from early stages on [14, 37–39]. Chronic kidney disease with of uremia and inflammation are also highly associated with biochemical constellation of GH resistance [40, 41]. In patients with sepsis and trauma, abnormal pH levels and cytokines are said to modulate the somatotropic axis, resulting in GH resistance [42]. This list can be further extended to cases of reduced insulin sensitivity up to poorly controlled insulin-dependent diabetes mellitus, where GH resistance has also been described [38]. Especially, in conditions characterized by development of cachexia such as chronic heart failure or chronic obstructive pulmonary disease, abnormalities in the IGF-I/GH axis can have a significant impact on diseases and prognosis [35, 43–46].This can also be applied this study cohort, who, with an average poor nutritional status and a measurably elevated risk of malnutrition and were carrying a crucial factor contributing to the development of a GH resistance.

Additionally, the average dependency on assistance, which is in this aged cohort likely caused by reduced muscle mass and function, suggests that muscle metabolism and, presumably, peripheral responsiveness to GH are altered [11].

Given that GH and IGF-I are potent anabolic mediators, resistance in these cases may lead to increased catabolism, impaired anabolism, and enhanced apoptosis, exacerbating a patient’s cachectic condition and worsening the prognosis [35]. Moreover, the GH resistance could potentially serve as both, either the cause or the consequence of these pathological conditions. That is why, in 2003 Cicoira et al. already stated that for example the chronic heart failure should not be considered merely a hemodynamic disorder, but moreover be viewed as a complex clinical syndrome with a hormonal component [35]. This perspective should be extended to other age-related conditions as well. Consequently, identifying factors that could enhance or restore GH/IGF-I function would offer additional treatment approaches for multimorbid patients with a coexisting GH resistance. This has the potential to improve the health of great parts of aging population significantly, given the high prevalence of the enumerated diseases above [29, 47]. However, the precise contribution of the different diseases to the endocrinologic disturbance remains unclear. Thus, there are also studies showing that, for example, besides presenting with growth hormone deficiency or GH resistance, laboratory mice indeed live longer than their counterparts [48]. Whether and to what extent a certain decline in the somatotropic axis may therefore be advantageous at a certain point of life remains unclear as well.

The effects of other contributing factors such as growth hormone binding hormone (GHBP), ghrelin, or somatostatin, could also cause disturbances in the somatotropic system, to an extend that remains unknown up to this date [49].

## Conclusion

Our research findings suggest that the pituitary gland, due to its remaining stimulation capacity in the oldest old, does not appear to be the sole cause of the age-related decline in the somatotropic axis during the somatopause.

Studies that uncover the exact underlying reasons for this IGF-I deficiency, be it a diminished hypothalamic activity, peripheral reasons or state of cachexia, are crucial for developing effective diagnostic and therapeutic approaches in the future.

## Data Availability

Detailed data can be made available upon request.
